# Increased normalized lactate load is associated with higher mortality in both sepsis and non-sepsis patients: an analysis of the MIMIC-IV database

**DOI:** 10.1186/s12871-022-01617-5

**Published:** 2022-03-25

**Authors:** Han Chen, Shu-Rong Gong, Rong-Guo Yu

**Affiliations:** Department of Critical Care Medicine, Fujian Shengli Clinical Medical College of Fujian Medical University, Fujian Provincial Hospital, No 134, Dongjie Street, Gulou District, Fuzhou, 350001 Fujian China

**Keywords:** Lactate, MIMIC-IV, Dynamic, Intensive care unit, Mortality

## Abstract

**Background:**

The present study aimed to evaluate the association between normalized lactate load, an index that incorporates the magnitude of change and the time interval of such evolution of lactate, and 28-day mortality in sepsis and non-sepsis patients. We also compared the accuracy of normalized lactate load in predicting mortality between these two populations.

**Methods:**

Data were extracted from the Medical Information Mart for Intensive Care (MIMIC)-IV database. We defined lactate load as the sum of the area under the lactate concentration curve; we also defined normalized lactate load as the lactate load divided by time. The performance of maximum lactate, mean lactate and normalized lactate load in predicting 28-day mortality in sepsis and non-sepsis patients were compared by receiver-operating characteristic curves analysis.

**Results:**

A total of 21,333 patients were included (4219 sepsis and 17,114 non-sepsis patients). Non-survivors had significantly higher normalized lactate load than survivors in sepsis and non-sepsis patients. The maximum lactate, mean lactate, and normalized lactate load AUCs were significantly greater in sepsis patients than in non-sepsis patients. Normalized lactate load had the greatest AUCs in predicting 28-day mortality in both sepsis and non-sepsis patients. Sensitivity analysis showed that the AUC of normalized lactate load increased in non-sepsis patients when more lactate measurement was obtained, but it was not improved in sepsis patients.

**Conclusions:**

Normalized lactate load has the strongest predictive power compared with maximum or mean lactate in both sepsis and non-sepsis patients. The accuracy of normalized lactate load in predicting mortality is better in sepsis patients than in non-sepsis patients.

## Introduction

Lactate is commonly used as an index of inadequate tissue perfusion and a marker to guide shock resuscitation in both sepsis and non-sepsis patients [1, 2]. Numerous studies are showing that elevated lactate is associated with increased mortality [3]. At a particular moment, a high lactate level is a “static” index reflecting the imbalance between its production and clearance, but it fails to reflect the change of lactate homeostasis. For this reason, some “dynamic” indices have been proposed to describe not only the magnitude but also the duration and trend of hyperlactatemia over time. Vincent et al*.* proposed serial lactate measurement in the early 1980s, and they found that survivors had a > 10% lactate reduction rate during the first 60 min of treatment [4]. The time variables in lactate kinetics continued to be studied in the following years, and lactate-guided treatment protocols were further advocated [5–11]. A recent systematic review that reviews all studies on lactate kinetics also suggests that decreasing lactate concentrations is consistently associated with better outcomes throughout the literature and applies to all situations of hyperlactatemia in heterogeneous patient populations [12].

New indices incorporating both the magnitude and the time interval of lactate change have been proposed since the 2010s [13–16]. The dynamic evolution of lactate is plotted against time, and the area under the curve (AUC) can thus represent the overall lactate burden, termed “lactate area” [14], “lactate area score” [15, 16], or “lactate load” [17]. By dividing the AUC by the time interval, one can obtain the averaged lactate load in this period, termed “time-weighted average lactate” [13] or “normalized lactate load” [17]. Such indices have been investigated mainly in sepsis patients and are associated with worse outcomes [14–16, 18]. Few studies include non-sepsis patients [13, 17]. Moreover, it is unclear whether there is a difference in diagnostic value between sepsis and non-sepsis population. We hypothesis that the performance of normalized lactate load in predicting mortality is different between the sepsis and the non-sepsis patients. The present study evaluated the association between normalized lactate load and 28-day mortality in sepsis and non-sepsis patients by analyzing data from a large critical care database. We also compared the accuracy of normalized lactate load in predicting mortality between these two populations.

## Materials and methods

### Database

Data were obtained from the Medical Information Mart for Intensive Care IV (MIMIC IV) database [19]. MIMIC-IV was published on March 16, 2021, as an update to the MIMIC-III database [20]. It contains de-identified health-related data associated with patients who stayed in critical care units of the Beth Israel Deaconess Medical Center between 2008 and 2019. Consent was obtained when the database was established and the original data was collected. Therefore, the Institutional Review Board of Fujian Provincial Hospital waived the informed consent for the present study. Dr. Han Chen and Dr. Shu-Rong Gong completed the online training course on database usage (certification number: HC 36014736, SRG 35606844) and extracted data. The study was designed and conducted under the Declaration of Helsinki.

### Data extraction

**Fig. 1:**
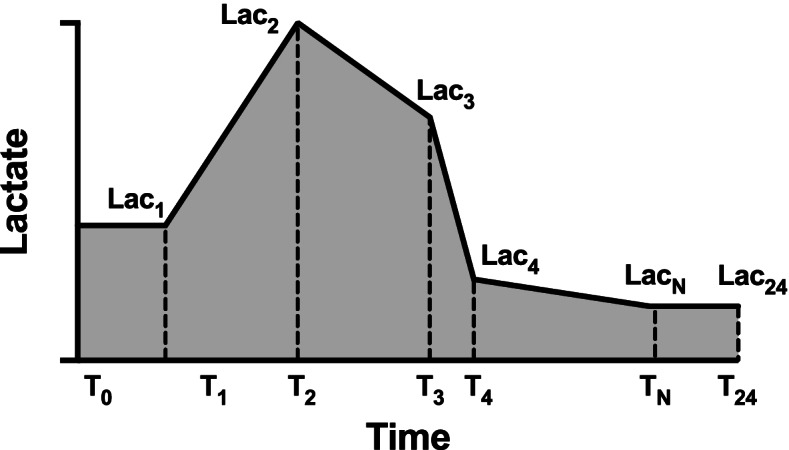
Diagram describing the calculation of lactate load and normalized lactate load. Each “T” on the x-axis represents the timepoint of lactate measurement, and “Lac” represents the corresponding lactate value. Lactate load was calculated as: (Lac_1_ + Lac_0_)/2 × (T_1_—T_0_) + (Lac_2_ + Lac_1_)/2 × (T_2_—T_1_) + … + (Lac_24_ + Lac_N_)/2 × (T_24_—T_N_). T_0_ represents the time of ICU admission, and the corresponding Lac_0_ was defined as equals to Lac_1_. Similarly, Lac_24_ (lactate value at 24 h after admission) was defined as equals to the last lactate value within the 24 h (Lac_N_). Normalized lactate load was calculated as lactate load divided by 24 h

The following data were extracted by PostgreSQL tools V.10.16 (PostgreSQL Global Development Group, CA, USA): age, gender, weight, comorbidities, the survival time, length of hospital stay, and length of ICU stay, sequential organ failure assessment (SOFA) score, simplified acute physiology score-II (SAPS-II), vital signs, first-day laboratory results, daily fluid input, fluid balance, and urine output. Besides, the time and value of lactate measurement in the first 24 h of ICU admission were also extracted to calculate the lactate-related parameters. We used “lactate load” to represent the cumulative effect of hyperlactatemia over time (i.e., the AUC of lactate) and “normalized lactate load” to describe the average intensity of hyperlactatemia (i.e., the quotient of AUC divided by time) as previously reported [18]. The calculation is detailed in Fig. 1.

### Study design

All patients admitted into ICU were screened. The exclusion criteria were: 1) age < 18 years; 2) not first ICU admission; 3) only one lactate measurement was obtained during the first 24 h; 4) length of ICU stay < 24 h. Patients were divided into the sepsis or non-sepsis group according to the sepsis-3.0 criteria [1].

### Statistical analysis

STATA (ver. 15.1, StataCorp., TX, USA) and MedCalc (ver. 15.8, MedCalc Software, Ostend, Belgium) were used for data analysis. Kolmogorov–Smirnov test was used to assess the normality of distribution. Continuous variables were presented as mean ± standard deviation or median with interquartile range according to the normality. Student’s *t*-test (for normal distribution) or Wilcoxon rank-sum test (for non-normal distribution) were used. Categorical variables were presented as counts (percentages) and the chi-square test was performed. Receiver-operating characteristic (ROC) curves were constructed to test the performance of maximum lactate, mean lactate, and normalized lactate load. The AUCs of the ROC curves were compared using the Delong test [21]. A *p* < 0.05 was considered significant.

## Results

**Fig. 2:**
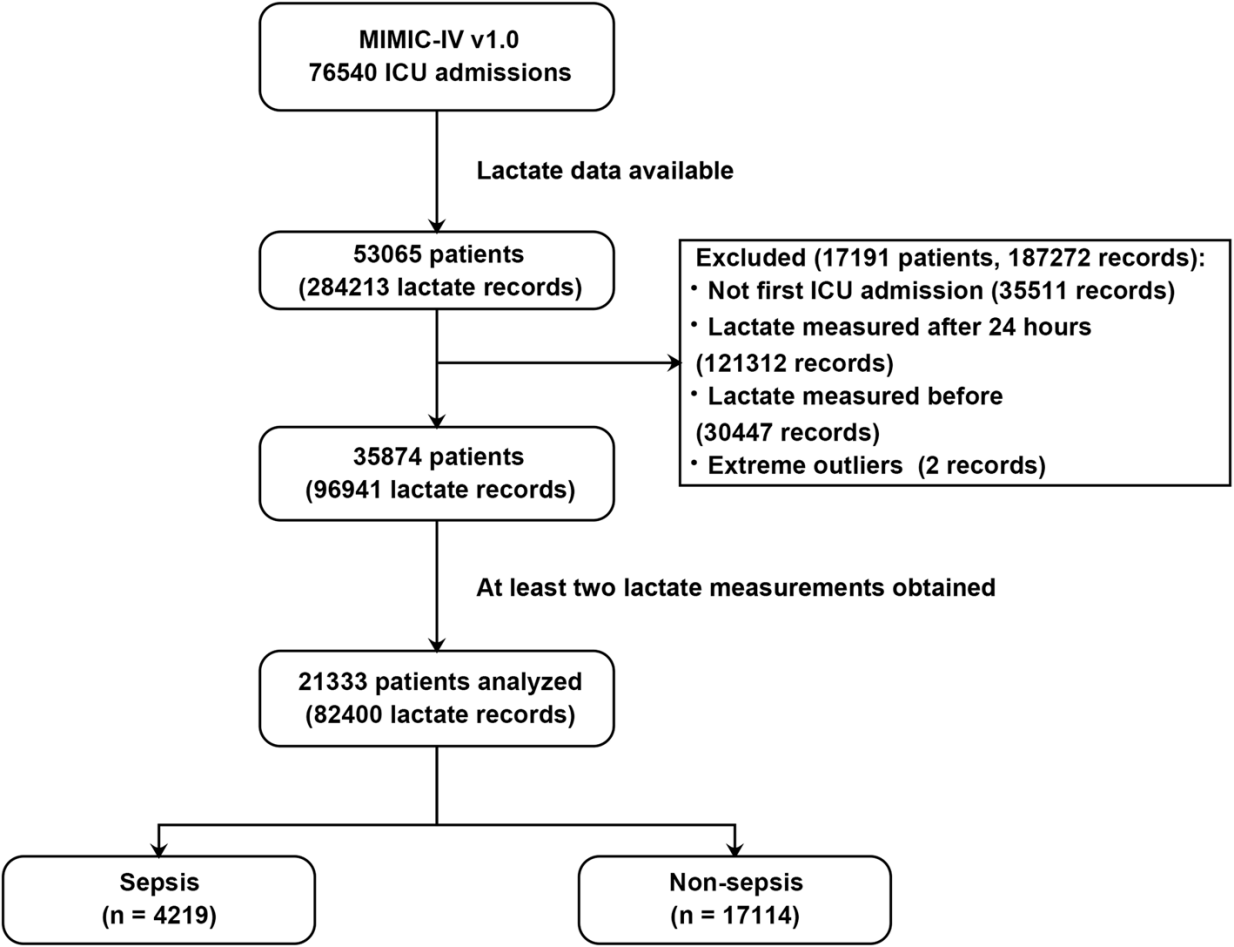
Flowchart showing a step-by-step selection of patients included in the study

**Fig. 3:**
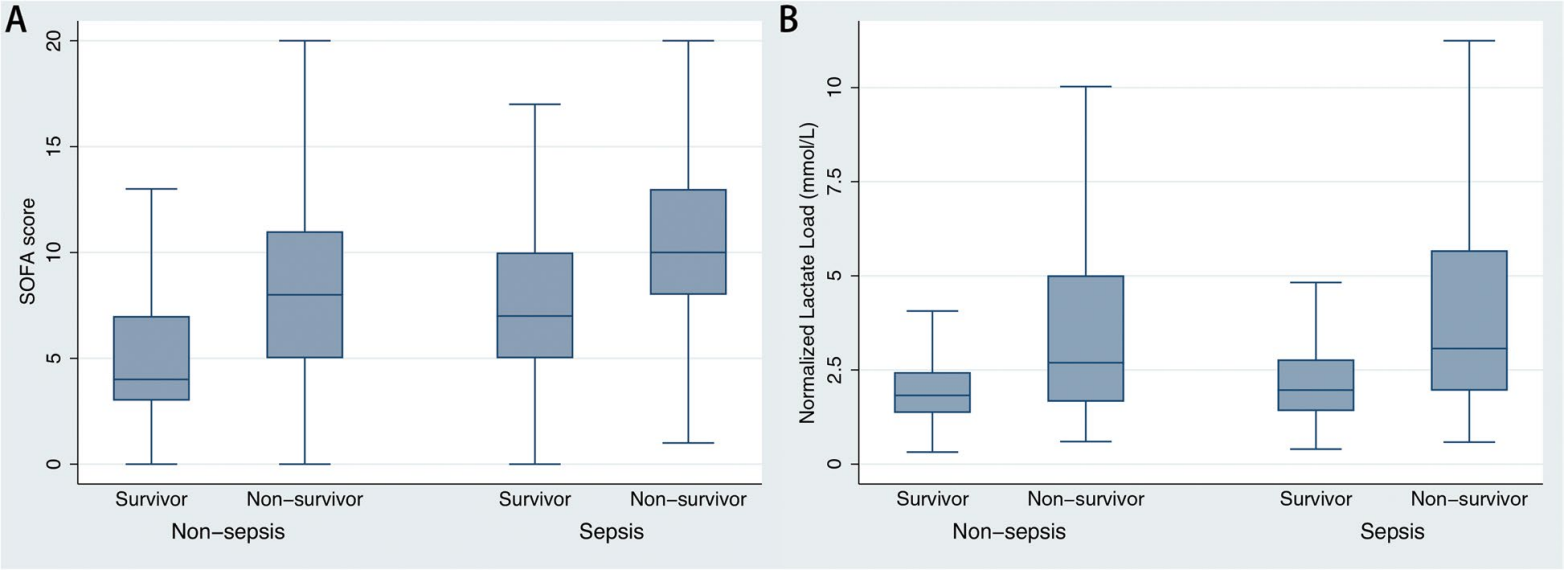
Severity score and normalized lactate load in sepsis and non-sepsis patients. Panel A: Sepsis patients had significantly higher sequential organ failure assessment (SOFA) scores than non-sepsis patients, while non-survivors had significantly higher SOFA scores than survivors in each group. Panel B: Similarly, sepsis patients had significantly higher normalized lactate load than non-sepsis patients, while non-survivors had significantly higher normalized lactate load than survivors in each group

A total of 21,333 patients were included (4219 sepsis and 17,114 non-sepsis patients, Fig. 2). Table 1 shows the baseline patient characteristics. In brief, sepsis patients were older, with higher severity scores (SOFA and SAPS-II, Fig. 3A), and were more likely to have underlying comorbidities. The 28-day mortality rate was significantly higher in sepsis patients than in non-sepsis patients (36.7% vs. 11.8%, *p* < 0.001). Sepsis patients had significantly length of hospital and ICU stay (9.8 [5.2, 17.8] *vs.* 7.8 [5.1, 12.6] days and 4.1 [2.1, 8.9] *vs.* 2.4 [1.4, 4.5] days, respectively; all *p* < 0.001). In addition, sepsis patients had significantly greater amount of fluid intake (2500 [1000, 4000], vs. 2000 [1000, 3000], *p* < 0.001) and less urine output (1030 [455, 1850] vs. 1615 [1010, 2420], *p* < 0.001) than non-sepsis patients in the first 24 h.

**Table 1:** Comparisons of the baseline clinical characteristics between sepsis and non-sepsis

	**Whole population (*n* = 21,333)**	**Non-sepsis (*n* = 17,114)**	**Sepsis (*n* = 4219)**	***p* value**
Age (year)	65.1 ± 16	64.5 ± 16.1	67.4 ± 15.4	< 0.001
Female (%)	8528 (40)	6650 (38.9)	1878 (44.5)	< 0.001
Weight (kg)	83 ± 23.8	83.1 ± 23.2	82.7 ± 26.2	0.233
SOFA score	5.9 ± 3.7	5.3 ± 3.3	8.6 ± 3.9	< 0.001
SAPS-II score	41.7 ± 15.1	39.6 ± 14.1	50.4 ± 15.9	< 0.001
Length of hospital stay (day)	8 (5.1, 13.5)	7.8 (5.1, 12.6)	9.8 (5.2, 17.8)	< 0.001
Length of ICU stay (day)	2.7 (1.4, 5.2)	2.4 (1.4, 4.5)	4.1 (2.1, 8.9)	< 0.001
24-h fluid intake (mL)	2000 (1000, 3250)	2000 (1000, 3000)	2500 (1000, 4000)	< 0.001
24-h urine output (mL)	1515 (891, 2330)	1615 (1010, 2420)	1030 (455, 1850)	< 0.001
Die in 28 days	3569 (16.7)	2019 (11.8)	1550 (36.7)	< 0.001
**Comorbidities**				
Congestive heart failure	6849 (32.1)	5308 (31)	1541 (36.5)	< 0.001
Myocardial infarction	4473 (21)	3629 (21.2)	844 (20)	0.086
Cerebrovascular disease	2302 (10.8)	1885 (11)	417 (9.9)	0.034
Chronic pulmonary disease	5602 (26.3)	4420 (25.8)	1182 (28)	0.004
Mild liver disease	3072 (14.4)	2106 (12.3)	966 (22.9)	< 0.001
Severe liver disease	1430 (6.7)	990 (5.8)	440 (10.4)	< 0.001
Diabetes without complication	5530 (25.9)	4361 (25.5)	1169 (27.7)	0.003
Diabetes with complication	2531 (11.9)	2020 (11.8)	511 (12.1)	0.579
Renal disease	4967 (23.3)	3766 (22)	1201 (28.5)	< 0.001
Malignant cancer	2473 (11.6)	1734 (10.1)	739 (17.5)	< 0.001
AIDS	159 (0.8)	108 (0.6)	51 (1.2)	< 0.001
**Laboratory results in the first 24 h**				
Maximum anion gap (mmol/L)	16.7 ± 5.7	16.1 ± 5.3	19.2 ± 6.3	< 0.001
Minimum albumin (g/dL)	2.9 ± 0.7	3.1 ± 0.7	2.7 ± 0.6	< 0.001
Maximum bilirubin (mg/L)	0.8 (0.4, 1.9)	0.7 (0.4, 1.6)	1.1 (0.5, 2.6)	< 0.001
Maximum creatinine (mg/dL)	1.7 ± 1.8	1.6 ± 1.7	2.3 ± 1.9	< 0.001
Maximum glucose (mg/dL)	196.7 ± 104.8	196.9 ± 102.4	195.9 ± 113.9	0.574
Minimum hemoglobin (g/dL)	9.4 ± 2.2	9.4 ± 2.2	9.4 ± 2.1	0.064
Minimum platelet (K/uL)	168 ± 97.5	166.1 ± 91.4	175.5 ± 118.7	< 0.001
Maximum potassium (mmol/L)	4.9 ± 0.9	4.9 ± 0.9	4.7 ± 0.9	< 0.001
Maximum APTT (sec)	47.3 ± 32.1	46.3 ± 31.9	51.2 ± 32.9	< 0.001
Maximum INR	1.7 ± 1.2	1.6 ± 1	2.1 ± 1.6	< 0.001
Maximum PT (sec)	18.8 ± 12.3	17.7 ± 10.6	23.1 ± 16.9	< 0.001
Maximum sodium (mmol/L)	140 ± 5.1	140.1 ± 4.7	139.7 ± 6.2	< 0.001
Minimum sodium (mmol/L)	135.5 ± 5.2	135.4 ± 4.9	135.7 ± 6.1	< 0.001
Maximum blood urea nitrogen (mg/dL)	30.2 ± 23.9	27.4 ± 21.8	41.5 ± 28.3	< 0.001
Maximum white blood cell count (K/μL)	16 ± 11.6	15.3 ± 10.5	18.6 ± 14.8	< 0.001
**Vital signs in the first 24 h**				
Mean heart rate (bpm)	87.6 ± 16	86 ± 15.2	93.7 ± 17.7	< 0.001
Minimum systolic blood pressure (mmHg)	85.6 ± 16.3	87.3 ± 16.1	78.5 ± 15.1	< 0.001
Minimum diastolic blood pressure (mmHg)	44.1 ± 10.8	45 ± 10.6	40.4 ± 10.7	< 0.001
Minimum mean arterial blood pressure (mmHg)	55.3 ± 14.1	56.7 ± 13.6	49.5 ± 14.4	< 0.001
Maximum respiratory rate (bpm)	28.9 ± 6.8	28.4 ± 6.6	31.2 ± 7.1	< 0.001
Minimum pulse O_2_ saturation (%)	91.1 ± 8.3	91.7 ± 7.4	88.6 ± 10.9	< 0.001
**Lactate related variables**				
Maximum lactate (mmol/L)	2.6 (1.8, 3.9)	2.6 (1.8, 3.7)	2.9 (1.9, 5.2)	< 0.001
Mean lactate (mmol/L)	2.1 (1.5, 2.9)	2 (1.5, 2.8)	2.3 (1.6, 3.7)	< 0.001
Lactate load (mmol·hr./L)	46.2 (33.8, 65.1)	45 (33.2, 61.8)	53.4 (37.1, 85.4)	< 0.001
Normalized lactate load (mmol/L)	1.9 (1.4, 2.7)	1.9 (1.4, 2.6)	2.2 (1.5, 3.6)	< 0.001

Data are presented as mean ± standard deviation or median (interquartile range) for continuous variables and counts (percentages) for categorical variables

*AIDS* Acquired immunodeficiency syndrome, *APTT* Activated partial thromboplastin time, *ICU* Intensive care unit, *INR* International normalized ratio, *PT* Prothrombin time, *SAPS-II* Simplified acute physiology score-II, *SOFA* Sequential organ failure assessment

Sepsis patients had significantly higher maximum lactate (2.9 [1.9, 5.2] *vs.* 2.6 [1.8, 3.7]mmol/L, *p* < 0.001), mean lactate (2.3 [1.6, 3.7] *vs.* 2 [1.5, 2.8] mmol/L, *p* < 0.001), lactate load (53.4 [37.1, 85.4] *vs.* 45 [33.2, 61.8]mmol·hr/L, *p* < 0.001), and normalized lactate load (2.2 [1.5, 3.6] *vs.* 1.9 [1.4, 2.6] mmol/L, *p* < 0.001) than non-sepsis patients. Non-survivors had significant higher normalized lactate load than survivors in sepsis (3.1 [2, 5.7] *vs.* 2 [1.4, 2.8] mmol/L, *p* < 0.001) and non-sepsis (2.7 [1.7, 5] *vs.* 1.8 [1.4, 2.4] mmol/L, *p* < 0.001) patients (Fig. 3B).

The AUCs of maximum lactate, mean lactate and normalized lactate load were significantly greater in sepsis patients than in non-sepsis patients (maximum lactate: 0.687 [95% confidence interval: 0.673—0.701] *vs.* 0.661 [0.654—0.668]; mean lactate: 0.697 (0.683—0.711) *vs.* 0.673 [0.666—0.680]; normalized lactate load: 0.707 [0.693—0.721] *vs.* 0.684 [0.677—0.691], all *p* < 0.001; Table 2). The AUC of normalized lactate load was also significantly greater than the AUCs of maximum lactate and mean lactate in the sepsis and the non-sepsis patients (all *p* < 0.001).

**Table 2:** Performance of normalized lactate load, maximum lactate, and mean lactate in predicting 28-day mortality

	**Cut-off value**	**AUC (95% CI)**	**Sensitivity (95% CI)**	**Specificity (95% CI)**	**Positive likelihood ratio (95% CI)**	**Negative likelihood ratio (95% CI)**	**Positive predictive value (95% CI)**	**Negative predictive value (95% CI)**
**All patients (*n* = 21,333)**								
Normalized lactate load	2.8	0.706 (0.700—0.712)	50.35 (48.7—52.0)	83.12 (82.6—83.7)	2.98 (2.8—3.1)	0.6 (0.6—0.6)	37.5 (36.1—38.9)	89.3 (88.8—89.8)
Maximum lactate	4	0.68 (0.674—0.686)	48.53 (46.9—50.2)	81.52 (80.9—82.1)	2.63 (2.5—2.7)	0.63 (0.6—0.7)	34.5 (33.2—35.9)	88.7 (88.2—89.2)
Mean lactate	3.2	0.694 (0.688—0.700)	46.15 (44.5—47.8)	85.9 (85.4—86.4)	3.27 (3.1—3.4)	0.63 (0.6—0.6)	39.7 (38.2—41.2)	88.8 (88.3—89.3)
**Sepsis (*n* = 4219)**								
Normalized lactate load	2.8	0.707 (0.693—0.721)	54.65 (52.1—57.1)	76.28 (74.6—77.9)	2.3 (2.1—2.5)	0.59 (0.6—0.6)	57.2 (54.7—59.8)	74.3 (72.7—76.0)
Maximum lactate	4.4	0.687 (0.673—0.701)	48.39 (45.9—50.9)	79.92 (78.3—81.4)	2.41 (2.2—2.6)	0.65 (0.6—0.7)	58.3 (55.6—61.0)	72.7 (71.1—74.3)
Mean lactate	3.3	0.697 (0.683—0.711)	47.74 (45.2—50.3)	81.57 (80.0—83.0)	2.59 (2.4—2.8)	0.64 (0.6—0.7)	60.1 (57.3—62.8)	72.9 (71.2—74.5)
**Non-sepsis (*n* = 17,114)**								
Normalized lactate load	3	0.684 (0.677—0.691)	43.54 (41.4—45.7)	87.88 (87.4—88.4)	3.59 (3.4—3.8)	0.64 (0.6—0.7)	32.5 (30.7—34.3)	92.1 (91.6—92.5)
Maximum lactate	4	0.661 (0.654—0.668)	46.16 (44.0—48.4)	82.52 (81.9—83.1)	2.64 (2.5—2.8)	0.65 (0.6—0.7)	26.1 (24.7—27.6)	92 (91.5—92.4)
Mean lactate	3	0.673 (0.666—0.680)	46.56 (44.4—48.8)	84.27 (83.7—84.9)	2.96 (2.8—3.1)	0.63 (0.6—0.7)	28.4 (26.8—29.9)	92.2 (91.7—92.6)

*AUC* Area under the curve, *CI* Confidence interval

### Sensitivity analysis

Sensitivity analysis was performed to test whether taking more lactate measurements can improve the accuracy of normalized lactate load in predicting mortality. We calculated the AUCs of ROC curves of normalized lactate load in patients who had 2, ≥ 3, ≥ 4, or ≥ 5 measurements of lactate within 24 h. The AUCs were not changed in sepsis patients. In contrast, AUCs increased in non-sepsis patients when more lactate measurements were obtained (changed from 0.684 to 0.775, Table 3).

**Table 3:** AUCs of normalized lactate load in patients with different number of lactate measurements

	**Sepsis**			**Non-sepsis**		
Number of lactate measurements	Number of patients	AUC	95% CI	Number of patients	AUC	95% CI
> = 2	4219	0.707	0.693—0.721	17,114	0.684	0.677—0.691
> = 3	2131	0.697	0.675—0.72	6778	0.734	0.714—0.753
> = 4	1501	0.687	0.66—0.714	4319	0.759	0.737—0.782
> = 5	1036	0.707	0.676—0.739	2810	0.775	0.749—0.8

*AUC* Area under the curve, *CI* Confidence interval

## Discussion

The main findings of this study were: 1) normalized lactate load had the strongest predictive power in both sepsis and non-sepsis patients; 2) normalized lactate load had better accuracy in sepsis patients than in non-sepsis patients; 3) the accuracy of normalized lactate load was improved when more lactate measurements were obtained in non-sepsis patients, while unchanged in sepsis patients.

Serum lactate concentration relates closely to the survival of ICU patients [2, 22, 23]. However, a single isolated lactate measurement is not good enough for predicting the outcome or guiding therapy. The magnitude of organ dysfunction depends upon the extent and duration of hypoxia. Since lactate can serve as a hypoxia marker, the production of the lactate value and the duration of hyperlactatemia can reflect the hypoxic burden [18]. In this regard, taking the dynamic change of lactate into account could provide more information and thus is attractive in managing critically ill patients [4, 8, 24].

“Lactate load” and “normalized lactate load,” first proposed by Zhang et al. [17], were used to describe lactate variation over time in post-cardiosurgical patients. We adopt these terms since “lactate load” can imply the concept of hypoxic load or hypoxic burden, and the term “normalized lactate load” thus reflects the “standardized” or the “averaged” hypoxic burden over time. The majority of the previous studies examined such an index (although with alternative nomenclature) in sepsis/septic shock [14–16]. However, there is a lack of comparison of the predictive power of this indicator between sepsis and non-sepsis populations. In one retrospective study involving a heterogeneous cohort of critically ill patients, Nichol and colleagues found that sepsis patients had an increased risk of mortality (odds ratio: 1.6), while no significant interaction was found between sepsis and time-weighted lactate in the multivariate model [13]. However, the ROC curves were not compared directly in their study. Our data suggest that normalized lactate load has moderate accuracy in predicting 28-day mortality in both sepsis and non-sepsis patients, and the accuracy is better in the sepsis population. In this regard, we provide new evidence to support the use of normalized lactate load in critically ill patients, especially in sepsis patients.

Our data suggest that the AUC and the sensitivity, specificity, negative predictive value, and positive predictive value of normalized lactate load were not very high. In other words, normalized lactate load is far from a perfect predictor of mortality. Indeed, a single indicator cannot accurately predict the prognosis in a highly heterogeneous population (i.e., the ICU patients) and should be integrated with various clinical manifestations, laboratory exams, and imaging.

Increasing the sampling frequency of a laboratory index can undoubtedly increase the possibility that the results reflect the “real state,” especially for an indicator that may have apparent fluctuations in the early stage of ICU stay (e.g., lactate). For this reason, we performed a sensitivity analysis to test whether taking more lactate measurements can improve the accuracy of normalized lactate load in predicting mortality. Interestingly, the AUC increased in non-sepsis patients when more lactate measurements were obtained, but it was not true in sepsis patients. One possible explanation is that there may be more determinants for mortality in sepsis patients (e.g., timely and adequate fluid resuscitation, effective source control, correct antibiotics, etc.). Besides, sepsis patients are more likely to receive catecholamine infusion. Catecholamine can promote glycolysis and increase lactate, which is not associated with tissue hypoxia in this condition [2]. Thus, the improvement in measurement accuracy cannot improve the predictive accuracy.

The present study has several limitations. First, the study was limited by the nature of the retrospective design and the data source. Second, there was not a standard protocol for lactate measurement in this study. Therefore, lactate load and normalized lactate load may be underestimated or overestimated. However, such results simply reflect the true effect of normalized lactate load measurement in real-world clinical practice. Third, one cannot distinguish a decreasing or increasing pattern of lactate kinetic change by calculating normalized lactate load.

## Conclusions

Normalized lactate load has the strongest predictive power compared with maximum or mean lactate in both sepsis and non-sepsis patients. The accuracy of normalized lactate load in predicting mortality is better in sepsis patients than in non-sepsis patients.

## Data Availability

The data supporting the findings of the current study are available from the MIMIC-IV database. However, these data were used under license, and restrictions apply to the availability of the data. Data are thus not publicly available but available from the corresponding author (Dr. Rong-Guo Yu) upon reasonable request and with permission of the holder of the database.
